# Malaria epidemiology in low-endemicity areas of the northern coast of Ecuador: high prevalence of asymptomatic infections

**DOI:** 10.1186/s12936-017-1947-0

**Published:** 2017-07-26

**Authors:** Fabián E. Sáenz, Andrea Arévalo-Cortés, Gabriela Valenzuela, Andrés F. Vallejo, Angélica Castellanos, Andrea C. Poveda-Loayza, Juan B. Gutierrez, Alvaro Alvarez, Yi Heng Yan, Yoldy Benavides, Luis Enrique Castro, Myriam Arévalo-Herrera, Sócrates Herrera

**Affiliations:** 1 Centro de Investigación para la Salud en América Latina, Escuela de Ciencias Biológicas, Pontificia Universidad Católica del Ecuador, Calle Pambacienda y San Pedro del Valle, Quito, Ecuador; 2 Caucaseco Scientific Research Center, Cali, Colombia; 3 Department of Mathematics, Institute of Bioinformatics, University of Georgia, Athens, GA USA; 4 Malaria Vaccine and Drug Development Center, Cali, Colombia; 5 Ministerio de Salud Pública, Guayaquil, Ecuador; 6 School of Health, Universidad del Valle, Cali, Colombia

**Keywords:** Asymptomatic malaria, Ecuador, Knowledge attitude and practices, Elimination

## Abstract

**Background:**

The recent scale-up in malaria control measures in Latin America has resulted in a significant decrease in the number of reported cases in several countries including Ecuador, where it presented a low malaria incidence in recent years (558 reported cases in 2015) with occasional outbreaks of both *Plasmodium falciparum* and *Plasmodium vivax* in the coastal and Amazonian regions. This success in malaria control in recent years has led Ecuador to transition its malaria policy from control to elimination.

**Results:**

This study evaluated the general knowledge, attitude and practices (KAP) about malaria, as well as its prevalence in four communities of an endemic area in northwest Ecuador. A total of 258 interviews to assess KAP in the community indicated that most people in the study area have a basic knowledge about the disease but did not use to contribute to its control. Six hundred and forty-eight blood samples were collected and analysed by thick blood smear and real-time PCR. In addition, the distribution of the infections was mapped in the study communities. Although, no parasites were found by microscopy, by PCR the total malaria prevalence was 7.5% (6.9% *P. vivax* and 0.6% *P. falciparum*), much higher than expected and comparable to that reported in endemic areas of neighbouring countries with higher malaria transmission. Serology using ELISA and immunofluorescence indicated 27% respondents for *P. vivax* and 22% respondents for *P. falciparum*.

**Conclusions:**

Results suggest that despite a great malaria reduction in Ecuador, transition from control to elimination would demand further improvement in malaria diagnostics, including active case detection to identify and treat parasite asymptomatic carriers, as well as community participation in its elimination.

## Background

**Fig. 1:**
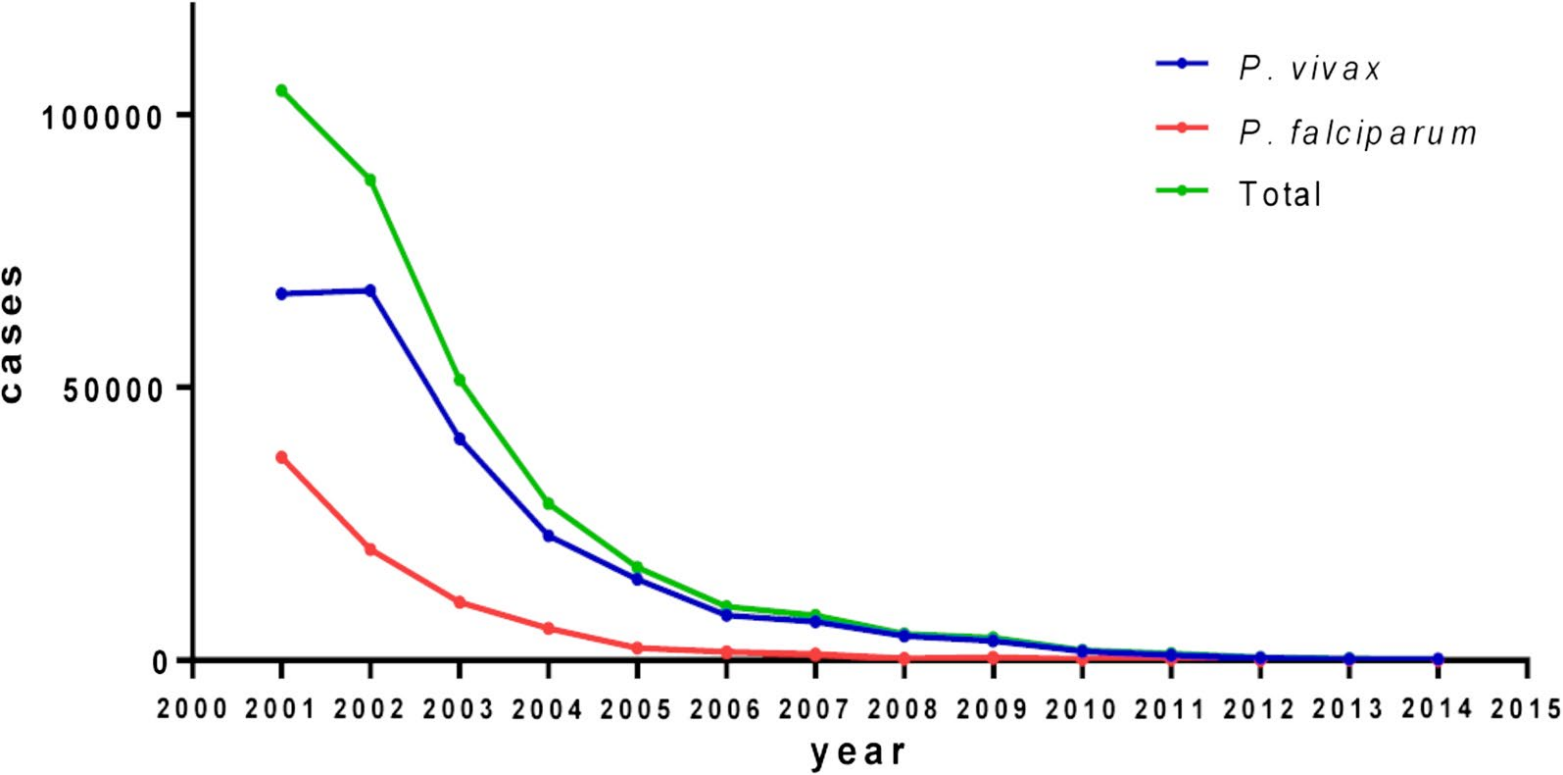
General trend of malaria in Ecuador 2001–2014. A 99% decrease in malaria cases is shown since the year 2001 for total cases (*green line*) as well as those caused by each of the main parasite species: *P. vivax* (*blue line*) and *P. falciparum* (*red line*)

Malaria is endemic in the coastal and Amazon regions of Ecuador in areas below 1500 m of altitude, however, the number of malaria cases has decreased >99% since 2001 and only 558 cases were reported in 2015 (Fig. 1); [1]. During the last 10 years, malaria control efforts have been highly effective and according to the Pan American Health Organization (PAHO), the country is currently in malaria pre-elimination phase [2, 3], closer to elimination than its neighbours Colombia and Peru. According to the World Health Organization, Ecuador is among the 21 countries that are in track for malaria elimination in 2020 [4]. Ecuador is focusing in fast detection of acute malaria clinical cases and treatment with artemether–lumefantrine (*Plasmodium falciparum*) and chloroquine + primaquine (*Plasmodium vivax*). Malaria surveillance activities are focused in the Northwest coast and in the Amazon regions, the current endemic areas [2].

Presently transmission is low and unstable with periodic outbreaks of both *P. falciparum* and *P. vivax*, still occurring on both sides of the Andes [1, 5]. The northwest coast of Ecuador, particularly Esmeraldas province, which limits on the North with the Nariño department of Colombia has historically been an area of *P. falciparum* transmission [5]. Although this region has experienced a substantial decrease in the number of reported malaria cases, it still maintains transmission and in the 2012–2013 period, it reported an outbreak of 150 *P. falciparum* cases in Esmeraldas city [5, 6]. In contrast, during the same period, San Lorenzo which historically had reported the highest *P. falciparum* annual parasite incidence (API) reported only ~20 cases [6]. In 2014 only 20 malaria cases were reported in Esmeraldas province but >100 cases were reported in both 2015 and 2016 [1].

Despite the low transmission, several challenges remain for Ecuador to progress towards the elimination goal. Major challenges are the accurate estimation of malaria prevalence, particularly in view of the number of asymptomatic cases potentially harbouring low levels of parasitaemia not detectable by microscopic examination and the instability of malaria control activities. As in many other regions, microscopic examination of thick blood smears is the gold standard for malaria diagnosis due to its good performance to confirm symptomatic infections. However, many infections have been shown to be submicroscopic and asymptomatic [7], leading to an underestimation of the number of infected individuals in endemic communities. These latter cases remain in the communities as parasite reservoirs for mosquito infection and require active case search as well as the use of more sensitive diagnostic methods. Although asymptomatic infections are considered to be the result of incremental immunity due to frequent exposure to parasite infections, and to reduce the disease burden, they contribute to maintaining malaria transmission [7–10]. Although a growing number of studies have estimated the prevalence of asymptomatic cases in diverse areas of Latin America such as Brazil, Colombia and Peru, as well as in regions in other continents with greater transmission intensities such as the Solomon Islands and Papua New Guinea (PNG) [7, 11–13], the proportion of asymptomatic malaria carriers in Ecuador has not been reported, leading to an underestimation of the real malaria incidence. This knowledge gap as well as others concerning the biology of vectors have to be addressed in malaria elimination programmes. Although *Anopheles albimanus* is considered the main vector species in northern Ecuador and southwest Colombia [14], the knowledge on its bionomy and potential vectors is limited [15, 16]. These knowledge gaps must be addressed for malaria elimination programmes.

The aim of this study was to better understand the epidemiological situation in the northern region of Ecuador, in view of the current malaria elimination plans. Four communities of Esmeraldas province were investigated by using sociodemographic and epidemiological surveys.

## Methods

### Study area

**Fig. 2:**
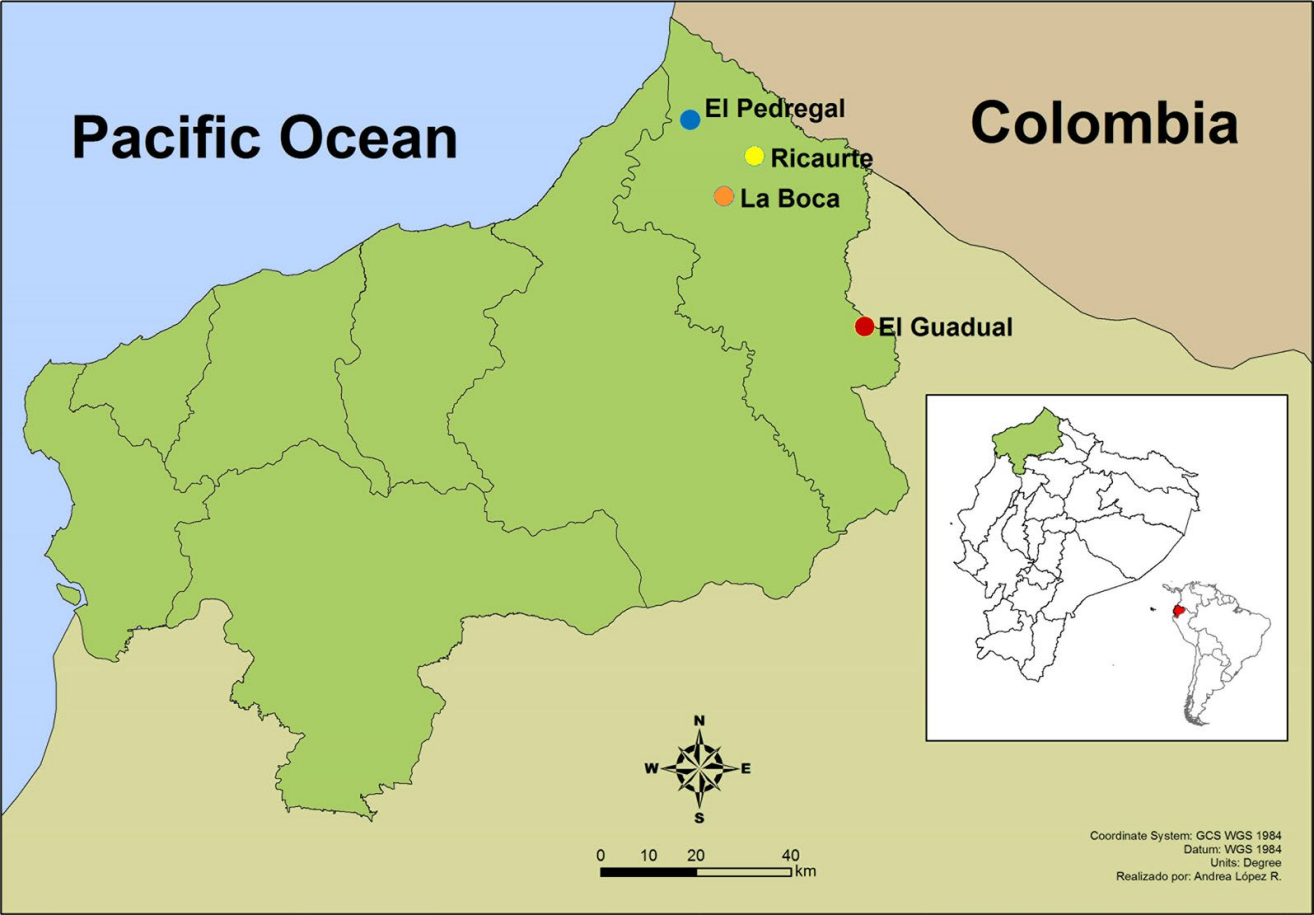
Study area. The study was performed in four locations in the Esmeraldas province (*green*) in Ecuador (*red*) which are indicated by *color dots* on the map

Four endemic settings in Ecuador’s San Lorenzo canton in Esmeraldas province were selected because of the relatively higher malaria prevalence, as compared to other parts of the country: El Pedregal, Ricaurte, La Boca, and El Guadual. All are located close to the road between Ibarra and San Lorenzo city (Fig. 2). *Plasmodium vivax* and *P. falciparum* are both transmitted in different proportions in these regions, which display an unstable transmission pattern. Pedregal is a semi-urban neighbourhood of the city of San Lorenzo, has a population of 270 inhabitants, and is located at sea level. Most inhabitants are described as African–Ecuadorians. The predominant malaria parasite species in this area is *P. falciparum* (six cases in 2013). Ricaurte is a semi-urban community in the Tululbí River 15 km southeast of El Pedregal; it has a population of 1300 inhabitants. La Boca is a rural community located 10 km southeast of Ricaurte at sea level, in the intersection of the Bogotá and Tululbí rivers; it has a population of 580 inhabitants predominantly African-Ecuadorian; El Guadual is a rural community located 40 km southeast of La Boca in the common border of Esmeraldas, Carchi, and Imbabura provinces; it is located at a higher altitude (600 m.a.s.l) and it has a population of 250 inhabitants predominantly mestizo; sporadic cases of *P. vivax* and *P. falciparum* have been previously reported in this community. A summary of the geographic and demographic characteristics of the study sites is shown in Table 1.

**Table 1:** Study communities: geographical and population characteristics

Locations	Altitude (m.a.s.l)	Average yearly rainfall (mm)	Average temperature (°C)	Population (inhabitants)	Community type	Dominant ethnicity
El Pedregal	9	2549	26.0	240	Semi-urban	African–Ecuatorians
Ricaurte	20	2553	25.9	1300	Semi-urban	African–Ecuatorians
La Boca	21	2474	26.0	580	Rural	African–Ecuatorians
El Guadual	600	2544	25.6	250	Rural	Mestizo

### Sample collection

A total of 649 individuals living in 258 houses of the study communities were studied. Blood samples were randomly chosen from residents of the four communities using the software Epidat to obtain a 95% confidence. Samples were collected in El Pedregal (177), Ricaurte (231), La Boca (165), and El Guadual (73). The blood was collected by finger prick and spotted on filter paper (Whatman 3MM) from inhabitants aged 2–70 years old. In addition, thin and thick blood smears were taken from each individual. All houses where georeferenced and geo-localized in a map.

### KAP evaluation

A simple KAP questionnaire was designed, approved by the Ethical Review Committees and used to survey a sample of the population. The questionnaire had a total of 13 questions: four questions of general knowledge about malaria transmission, three about prevention of malaria and six about malaria history.

### Ethical statement

The study protocol was approved by the Ethical Review Committees of Pontificia Universidad Catolica del Ecuador (# CBE-016-2013). Written informed consent (IC) was provided by study participants and/or their legal guardians (informed assent) for both the KAP survey and sample collection.

### Parasitological tests

#### Microscopy

The thin and thick blood smears taken from each individual were stained by the standard Giemsa method and used to confirm malaria infection [17]. Parasite densities were determined by using an optical microscope with 100× magnification and counting the number of parasites present in 400 white blood cells (WBC) at different fields and read by two independent microscopists. The total number of parasites per μl was estimated assuming patients had a WBC count of 8000/μl. Thin smears were used for *Plasmodium* species confirmation.

#### Real time PCR (qPCR)

DNA was extracted using the PureLink Genomic DNA Mini Kit (Life Technologies, USA), and real time PCR was performed as previously described [13, 18, 19], using primers targeting the 18S rRNA gene. Standard *P. falciparum* and *P. vivax* DNA positive and negative controls were used in each batch of tests including the extraction of both negative and inhibition control. A sample was considered negative if there was no increase in the fluorescent signal after a minimum of 40 cycles. Parasitaemia quantification was performed using a parasite specific standard curve made with serial blood dilutions of a reference field isolate. Each reaction plate included a standard curve for parasite quantification and positive results were independently confirmed. All positive results were reported to the local health authorities.

### Serological assays

The serum was extracted from blood samples on filter paper as previously described [20]. The dried samples were cut out (two circles of 3 mm), placed in 1.5 ml tubes and eluted in a stationary manner in 1 ml of PBS/0.05% Tween 20 overnight at 4 °C. Eluted serum was used to determine anti-malarial antibodies using both indirect immunofluorescence (IFAT) and enzyme linked-immunofluorescence assay (ELISA) methods.

For IFAT, *P. falciparum* or *P. vivax* infected red blood cells were used to produce antigen slides, which were processed as previously described [21]. Serum samples were incubated at 1:10 or 1:20 dilution and labelled with fluorescein isothiocyanate (FITC) conjugated-affinity goat–human IgG antibody at 1:1500 dilutions. Slides were examined under an epifluorescence microscope. For ELISA both *P. vivax* and *P. falciparum* antigens were used as follows: for *P. vivax* a long synthetic peptide corresponding to the amino terminus of the circumsporozoite (CS) protein (CS-N) and an amino terminal recombinant fragment (r200L) of the merozoite surface protein 1 (MSP-1); for *P. falciparum* a synthetic fragment corresponding to the amino flank (CS-N fragment) was used. These antigens have been previously shown to be highly immunogenic [22–25]. IgG against pre-erythrocytic *P. vivax* and asexual blood stages antigens of both *P. vivax* and *P. falciparum* were determined by ELISA as described elsewhere [22]. Briefly, 96-well plates (Nunc-Immuno Plate, Maxisorp, Roskilde, Denmark) were coated overnight with recombinant or synthetic protein fragments at 1.0 μg/ml and then blocked with 5% skim milk in PBS 1X, 0.05% Tween20 (PBS-T) for 1 h at room temperature. Test sera diluted 1:200 in 2.5% skim milk in PBS-T were then incubated for 1 h at 37 °C. After washing, the plates were incubated for 1 h at 37 °C with a 1:1000 or 1:2000 dilution of alkaline phosphatase-conjugated goat anti-mouse/human IgG antibody (Sigma Chemical Co., St Louis, MO). Cut-off points for ELISA were calculated as three SD above the mean absorbance value at 405 nm of normal human sera from healthy adult volunteers who had never been exposed to malaria. The results were also expressed as reactivity index (RI) defined as OD values of tested samples divided by the cut-off value.

### Statistical analysis

#### Data entry

Study data were collected and managed using REDCap electronic data capture tools hosted at the Caucaseco Research Center, Cali, Colombia [26]. REDCap (Research Electronic Data Capture) is a secure, web-based application designed to support data capture for research studies, providing: (1) an intuitive interface for validated data entry; (2) audit trails for tracking data manipulation and export procedures; (3) automated export procedures for seamless data downloads to common statistical packages; and (4) procedures for importing data from external sources. Data analysis was conducted with MATLAB release 2012b. Chi square statistical test was used to categorize malaria knowledge and practices, antibody frequencies and the parasitological prevalence.

### Spatial analysis of malaria cases in the study area

To generate a smooth representation of the discrete malaria case data collected during the study, a Gaussian kernel estimator was used to produce a population density map, a disease incidence map and a disease risk map per each village in the study. The population data of each village was smoothed using a Gaussian kernel estimator where the surveyed population data per household were treated as samples (X_1_; X_2_, …, Xn) drawn from some unknown distribution f(y), where y is a geographical coordinate. X_i1_ is the latitude of the sample and X_i2_ is the longitude of the sample. To estimate the shape of this distribution f, we used a Gaussian kernel with bandwidth h_1_ of 0.0005 longitude and h_2_ of 0.0005 latitude. Where the density of the function f(y) is estimated using the following expression:

$$f\left( y \right) = \frac{1}{n}\mathop \sum \limits_{i = 1}^{n} \frac{1}{{h_{1} }}\frac{1}{{h_{2} }}K\left( {\frac{{y_{1} - x_{i1} }}{{h_{1} }}} \right)K\left( {\frac{{y_{2} - x_{i2} }}{{h_{2} }}} \right).$$

Before producing the contour plot of the smoothed population data, a convex hull was produced to estimate the area of each village. The disease incidence data per each village was also smoothed using a Gaussian kernel estimator and a contour plot was produced and cropped using the same convex hull for the population distribution of each village. Finally, the disease risk map was produced by dividing the value of each point in the disease incidence map by the value of each point in the population distribution map.

## Results

### KAP survey

The general malaria KAP survey was performed on a sample of the population (n = 258 houses), and one person was surveyed in each house. In terms of knowledge, it was found that only a small percentage of the population (5–10%) see malaria as the main health problem that affects their communities when compared to other diseases such as respiratory diseases. In addition, 50–75% of the surveyed people in the communities declared to know how malaria is transmitted and 90–100% knew that a mosquito is responsible for malaria transmission. Likewise, most surveyed people (60–75%) correctly identified the main symptoms of malaria such as fever and headache. Only in El Guadual, most families identified chills as an important symptom. This was significantly different from the other three communities (*P* = 0.02). Regarding malaria prevention practices, >90% of the surveyed families identified the use of bed nets and closing doors and windows as the main activities to prevent malaria, while other activities such as use of mosquito repellent and insecticide-treated bed nets were not as commonly recognized except by families in El Pedregal (*P* = 0.04). A small proportion of the families (10–30%) indicated to take actions against malaria outside of the house like cleaning grass and bushes.

### Prevalence of *Plasmodium* infections

Six hundred and forty-eight thick and thin blood smear slides from the four study communities were analysed by optical microscopy. All were negative for the presence of *Plasmodium* parasites, however, when analysed by qPCR a mean overall prevalence of 7.4% *Plasmodium* infections was confirmed in the study area. The positivity rate for each community was 13% for El Pedregal, 2% for Ricaurte, 10% for La Boca and 5% for El Guadual. Among the qPCR positive volunteers, only one presented symptoms resulting in 98% asymptomatic infections.

**Fig. 3:**
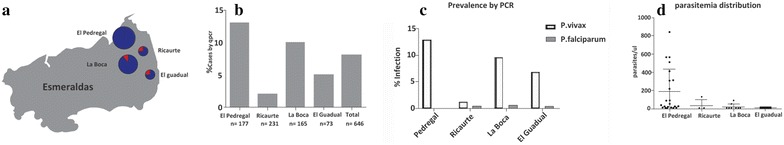
*Plasmodium* prevalence in the study area by qPCR. **a** Distribution of malaria infections in the study area. The *size of the circles* represents malaria prevalence, with *P. vivax* in *blue* and *P. falciparum* in *red*. **b** Percentage of *Plasmodium* cases by qPCR in the four study communities. El Pedregal had the highest prevalence while Ricaurte had the lowest. **c** *P. vivax* and *P. falciparum* prevalence. *P. vivax* was the most prevalent parasite in the study area. El Guadual had the highest amount of *P. falciparum* infections. **d** Parasitaemia distribution in the four study communities. Parasitaemia varied between 1 and 800 parasites/µl

The most prevalent parasite was *P. vivax* with 6.9% prevalence in the study communities (13% in El Pedregal, 1.3% in Ricaurte, 9.7% in La Boca, and 4.1% in El Guadual; Fig. 3). The parasitaemia levels varied between 1 parasite/µl and 813 parasites/µl. Among the four study populations, El Pedregal had a mean parasitaemia of 131.7 parasites/µl (SD: 179.6), Ricaurte 36.75 (SD: 62.19), La Boca 16.59 parasites/µl (SD: 23.37) and El Guadual 15 parasites/µl (SD: 6.73) (Fig. 3). El Guadual had the highest proportion of *P. falciparum* infections (1.4%).

### Antibody responses

**Fig. 4:**
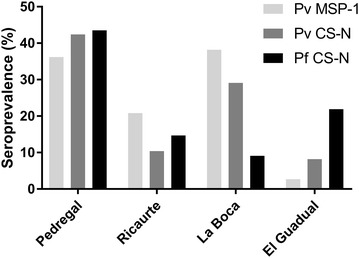
Percentage of antibody responders by ELISA. Percentages of antibody respondents to the indicated malaria antigens are shown as *bars* for the indicated study sites. The antibody response percentages broadly correlated with the malaria prevalence in each of the study sites. Indeed, El Pedregal had a higher percentage of responders for all antigens and a higher malaria prevalence

A total of 646 individuals were evaluated for antibody levels against both parasite species. Overall, 48.77% of volunteers presented antibodies against antigens of either *P. vivax* or *P. falciparum*. Specifically, 23.08% of volunteers presented antibodies against *Pv*CS-N, 27.23% against *Pv*MSP1 (*Pv*r200L) and 21.69% against *Pf*CSN. The community with a higher percentage of responders for all three antigens was El Pedregal (42% responders for *Pv*CS-N, 36% responders for *Pv*MSP1 and 44% responders for *Pf*CS N), followed by La Boca (29% responders for *Pv*CSN, 38% responders for *Pv*r200L and 9% responders for *Pf*CS-N), and Ricaurte (10% responders for *Pv*CSN, 21% responders for *Pv*r200L and 14% responders for *Pf*CS-N). El Guadual had the lowest percentage of responders against two of the three antigens (8% responders for *Pv*CS-N, 3% responders for *Pv*MSP1 and 22% responders for *Pf*CS-N) (Fig. 4).

When looking at specific antigens for *P. vivax* and *P. falciparum*, there was an overall seroprevalence of 11% of respondents to both *P. vivax* antigens tested and a 9.4% of responders to the *P. falciparum* antigen CS-N. The community with the highest antibody prevalence was El Pedregal while the community with the least prevalence was El Guadual (Fig. 4). The IFAT performed with samples PCR positive for *Plasmodium* indicated 12.5% positive samples (Table 2).

**Table 2:** IFA positivity in PCR positive samples

Location	n = (PCR+)	IFA positive	%
El Pedregal	23	22	96
El Guadual	4	2	50
Ricaurte	4	4	100
La Boca	17	14	82
Total	48	42	87.5*

* Average percentages

### Malaria distribution map in study populations

**Fig. 5:**
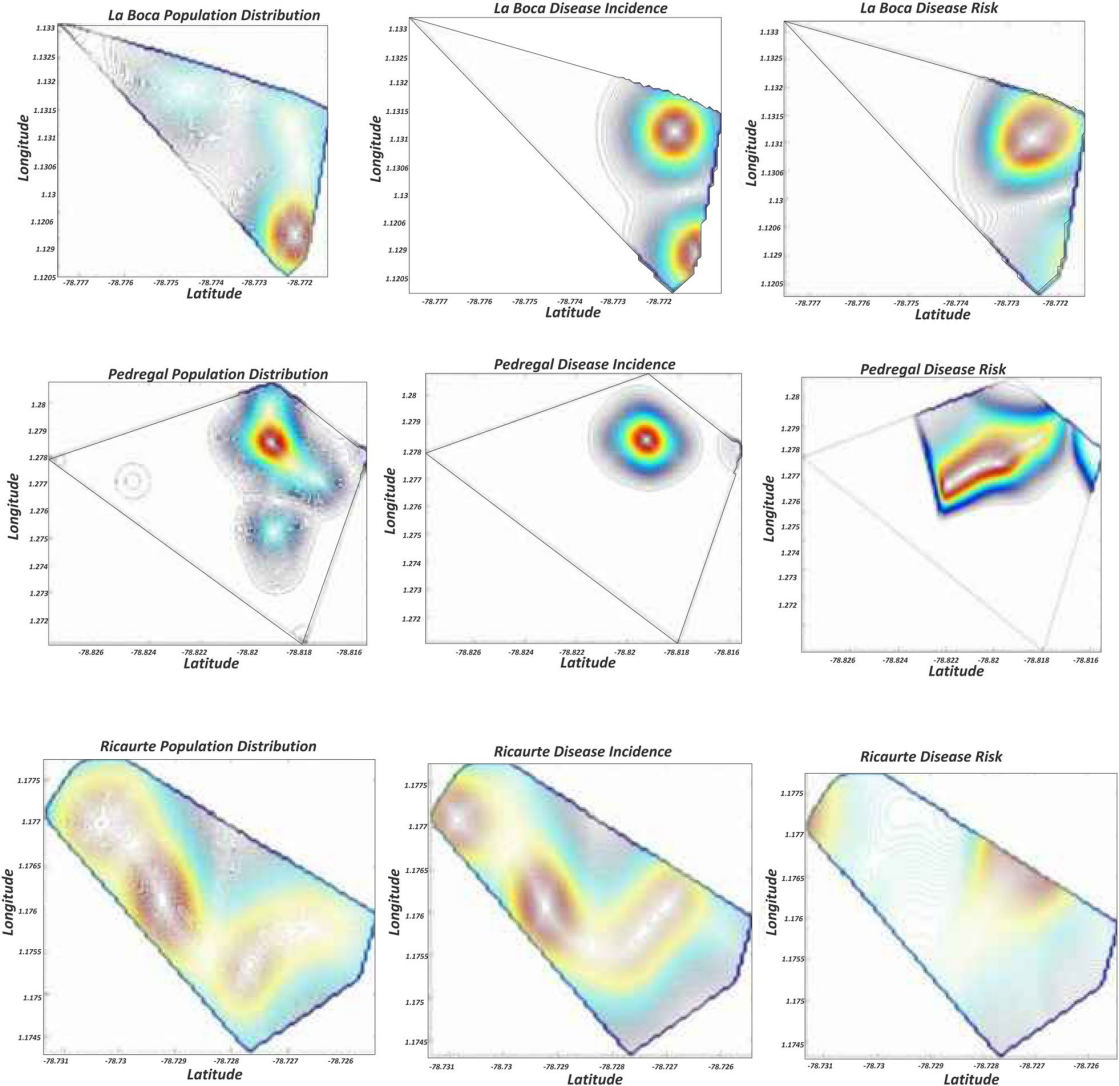
Estimated risk of malaria for La Boca (*top row*), El Pedregal (*middle row*), and Ricaurte (*bottom row*). The risk maps (*right column*) show the correlation between human population density and malaria cases, thus identifying the places where interventions might have the greatest impact. El Guadual was not included in this analysis because the number of samples was too small to perform the algorithm

The human population distribution was used alongside with the case distribution to generate a geographical representation of malaria risk in El Pedregal, Ricaurte and La Boca (the number of surveyed houses in El Guadual was too small to generate a significant map). In La Boca there was a high concentration of malaria in places where the population density was low, thus clearly delineating an area of high risk (Fig. 5); this could suggest localized anopheline breeding sites, or a concentration of inhabitants with additional risk factors (e.g. occupational). In El Pedregal, malaria cases were more frequent in areas of higher human population density, thus, the risk was closely proportional to the human population. In Ricaurte, there was an area of low population density and high number of cases, thus creating a zone of increased risk as compared to the rest of the locality.

## Discussion

Ecuador has experienced ~99% reduction in malaria clinical cases in the last 15 years and is now pointing towards an elimination phase. Despite periodic focal outbreaks, the country has shown a malaria transmission decreasing trend. In 2014, Ecuador reported the lowest malaria incidence of the last decade but in 2016 the reported incidence (917 cases), almost doubled that of the previous year. The study indicated the presence of a significant number of asymptomatic malaria parasite carriers in the study sites, with parasitaemia detectable only by qPCR which is probably an indication of an official under reporting of cases.

The periodic outbreaks, particularly the most recent one, appear to depend on several features. First, as most countries do, the National Malaria Control Programme (NMCP) of Ecuador uses microscopy and more recently RDT for malaria diagnosis. Although these two methods have great value for passive case detection of symptomatic cases, they show very limited sensitivity to detect asymptomatic cases which frequently display submicroscopic parasitaemia levels. Second, the intense population mobility from and to Colombia, may contribute to frequent reintroductions of malaria parasites. These and other issues may slow down the elimination process.

In the last several years there has been an increasing number of studies indicating the presence of asymptomatic submicroscopic malaria cases in all endemic areas of the world; unfortunately, in Ecuador there is a lack of studies addressing the malaria prevalence with more sensitive methods. Indeed, in contrast with the official records, this study confirmed a prevalence of asymptomatic infections between 2 and 13% in the different study sites, greater than expected and with a higher rate of *P. vivax* (6.9%). The study also indicated a good level of malaria knowledge in the study communities, however, the surveyed population declared not to be participating significantly in control activities, even though the contribution of the endemic communities as well as that of several other malaria stakeholders to malaria elimination is considered essential.

Despite the fact that moving from malaria control to elimination, may appear as simple as intensifying control activities, elimination requires a significant sustained effort with critical strategic programme changes to ensure the goal of reaching zero cases.

Because the NMCP does not usually seek identification of asymptomatic infections, they frequently remain undiagnosed, and even if they are searched, thick smears have limited sensitivity. Furthermore, there is no policy about treatment of these infections, and therefore asymptomatic carriers may be contributing to maintaining the parasite transmission to *Anopheles* as suggested by previous studies [7, 9, 26]. In areas with low transmission levels, asymptomatics could be critical reservoirs in the maintenance of transmission, thus hampering elimination. The reported 7.5% prevalence by PCR in the study areas was higher than that reported for the northern coast of Peru (1.2%) [20], but comparable to the prevalence recently found in the Colombian border (9.5%) [7] and the Peruvian Amazon (10.9%) [27], suggesting that even though the number of reported cases in Ecuador is lower than on the Colombian border, the transmission level may be similar. This finding is of great importance because, officially, Colombia is considered to be one of the countries with greater transmission in the Latin America region, while Ecuador is among those in pre-elimination phase. However, these results are similar to those reported previously from the Colombian coast, where PCR detected 26 times more parasites than microscopy when reactive case detection was used [7]. These results underscore the shortcomings of microscopy as a diagnosis technique and indicate that active surveillance in the area by the NMCP may not be enough to attain elimination. Moreover, all reported cases in this study were submicroscopic despite parasitaemia varying between 1 and 800 parasites/μl by PCR. It is likely that the amount of submicroscopic infections could be even higher because the PCR used here has a detection limit of ~2 parasites/μl [7]. In this study, a real time PCR was used due to the convenience of the DNA transportation in filter paper. More sensitive methods such as RNA reverse transcriptase could have been used but the inability to preserve RNA was a limitation in the study setting.

On the other hand, the presence of asymptomatic cases indirectly indicates that despite the low transmission intensity the community has developed substantial levels of clinical immunity, even though overall, less than one-third of the population displayed positive titers to both parasite species. Even in El Pedregal where the highest reactivity was recorded, less than half of the study population was positive. The seroreactivity found was in general low, but the frequency of seropositive individuals appeared to correlate with the malaria prevalence, it was higher in El Pedregal which displayed greater number of submicroscopic infections. While this study used a basic serological approach, more information could have been obtained from an analysis of age dependent antibody response and IFAT in all the studied population.

It is surprising that, although Esmeraldas province is the only area of Ecuador still regularly reporting *P. falciparum* cases, in this study, *P. vivax* was by far the most prevalent parasite (94% of infections against only 6% caused by *P. falciparum*). In this province only four cases of *P. vivax* were reported in 2013 [6]. This finding might be explained by the high prevalence of asymptomatic *P. vivax* infections only detectable by q PCR, and is in contrast with a greater prevalence of *P. falciparum* reported in southern Colombia during the same period [7, 28]. A possible explanation for this could be related to the fact that a large part of the population may express reduced Duffy antigens on the surface of their RBCs as a result of heterozygous genotypes as has been shown in other populations that allow *P. vivax* infections at low parasitaemia and develop immunity against this parasite [29], consistent with the frequency of antibodies to *P. vivax* in El Pedregal and La Boca.

The high prevalence of *P. vivax* in an area where it is rarely reported brings new challenges from the treatment point of view. Ecuador uses a radical cure treatment that includes a 3-day chloroquine course and a 7 day primaquine treatment. The need for primaquine treatment for *P. vivax,* in an area not previously reported, will have important consequences in a possible elimination setting, considering that the status of glucose-6-phosphate dehydrogenase (G6PD) deficiencies is uncertain. Even though *P. vivax* treatment has been performed without G6PD deficiency testing in Ecuador, models predict that the north of the country, an area where *P. vivax* is rarely reported has a higher prevalence of G6PD deficiency than other areas where vivax infections are more common [30]. As elimination approaches, the treatment of asymptomatic *P. vivax* patients will need to go hand in hand with the determination of the G6PD status for each patient.

The communities of El Pedregal, Ricaurte and La Boca with a greater historical exposure to malaria presented better knowledge about malaria but comparatively lower than in the adjacent coast of Colombia [31]. In contrast, in El Guadual, a community located towards the mountain side there was a lack of knowledge about malaria and no prevention practices were identified, this may correspond to recent introduction of malaria at these higher altitudes. Indeed, antibodies in this community indicated a lower previous exposure to the parasite than in the lowland study sites.

Although most people had a basic knowledge about malaria, practices to prevent malaria transmission were not very common. However, geo-referencing of malaria cases allowed mapping specific disease foci which facilitate targeting by control and elimination activities including education and community participation.

## Conclusions

Taken together, the results of this study demonstrate higher malaria prevalence than expected in the north coast of Ecuador that may be related to environmental factors, with most cases corresponding to *P. vivax* asymptomatic and submicroscopic infections. Although this may be due to significant clinical immunity, it evidences diagnosis challenges for the NMCP and in addition may be contributing to the maintenance of transmission. The elimination goal in Ecuador would imply important changes including more sensitive diagnostic methods and active case detection, and probably community participation to eliminate mosquito breeding places among others.
